# Supplementary material for: Pitfalls in AR42J-model of cerulein-induced acute pancreatitis
Source: PLoS One. 2021 Jan 25;16(1):e0242706. doi: 10.1371/journal.pone.0242706 (PMC7833168; doi:10.1371/journal.pone.0242706)

Figure 2 A1

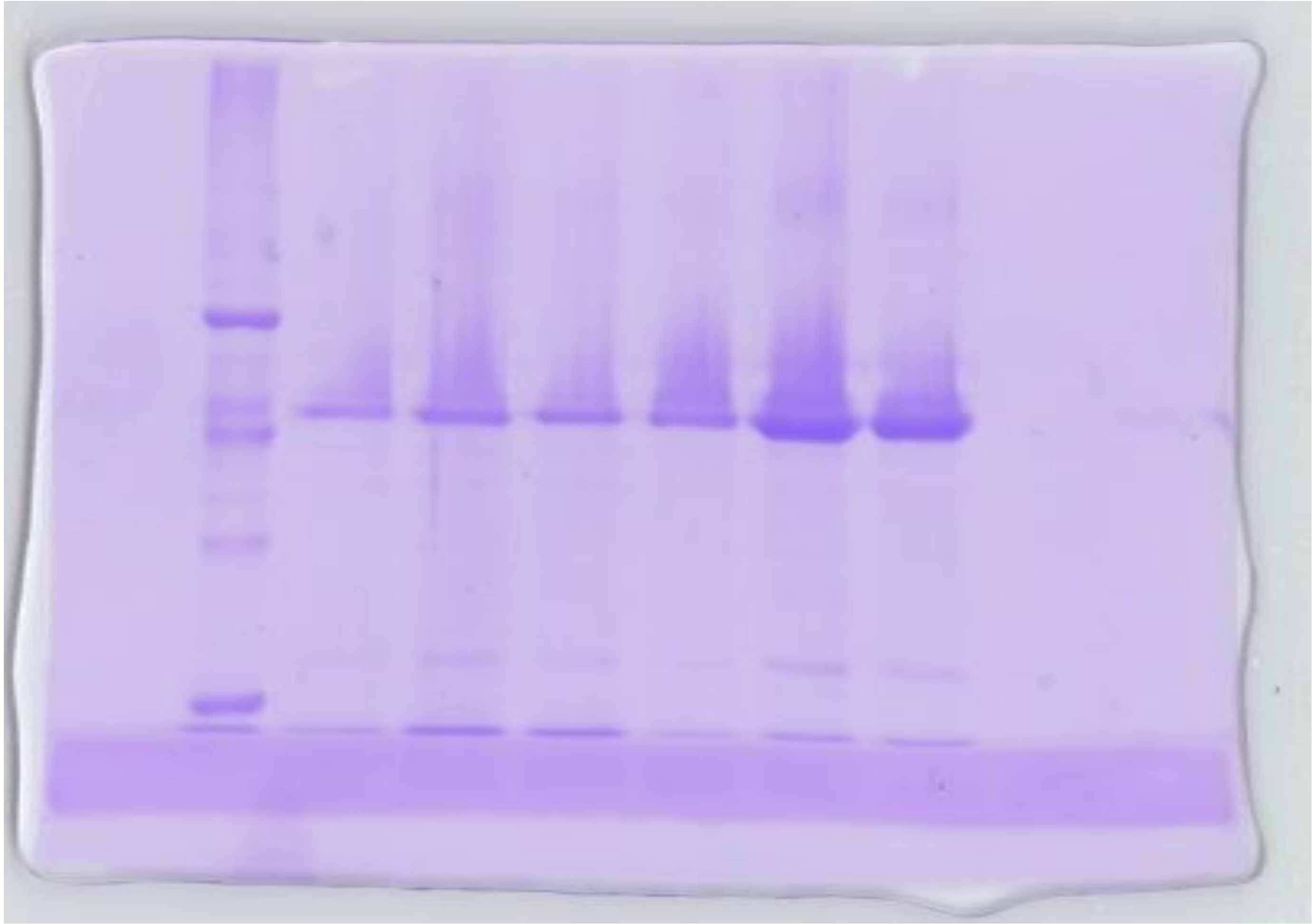

Figure 2 E1
Amylase

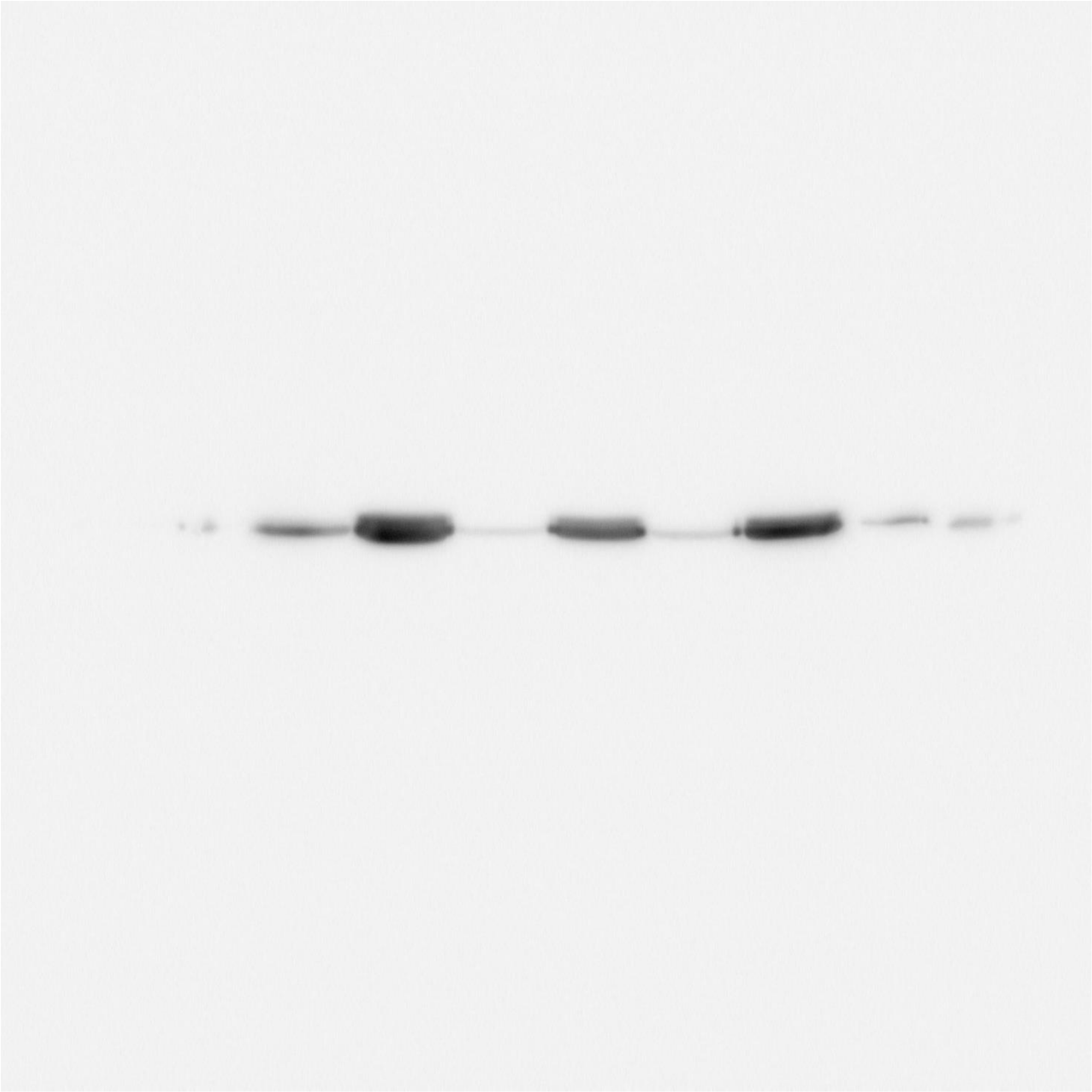

Figure 2 E1
Lipase

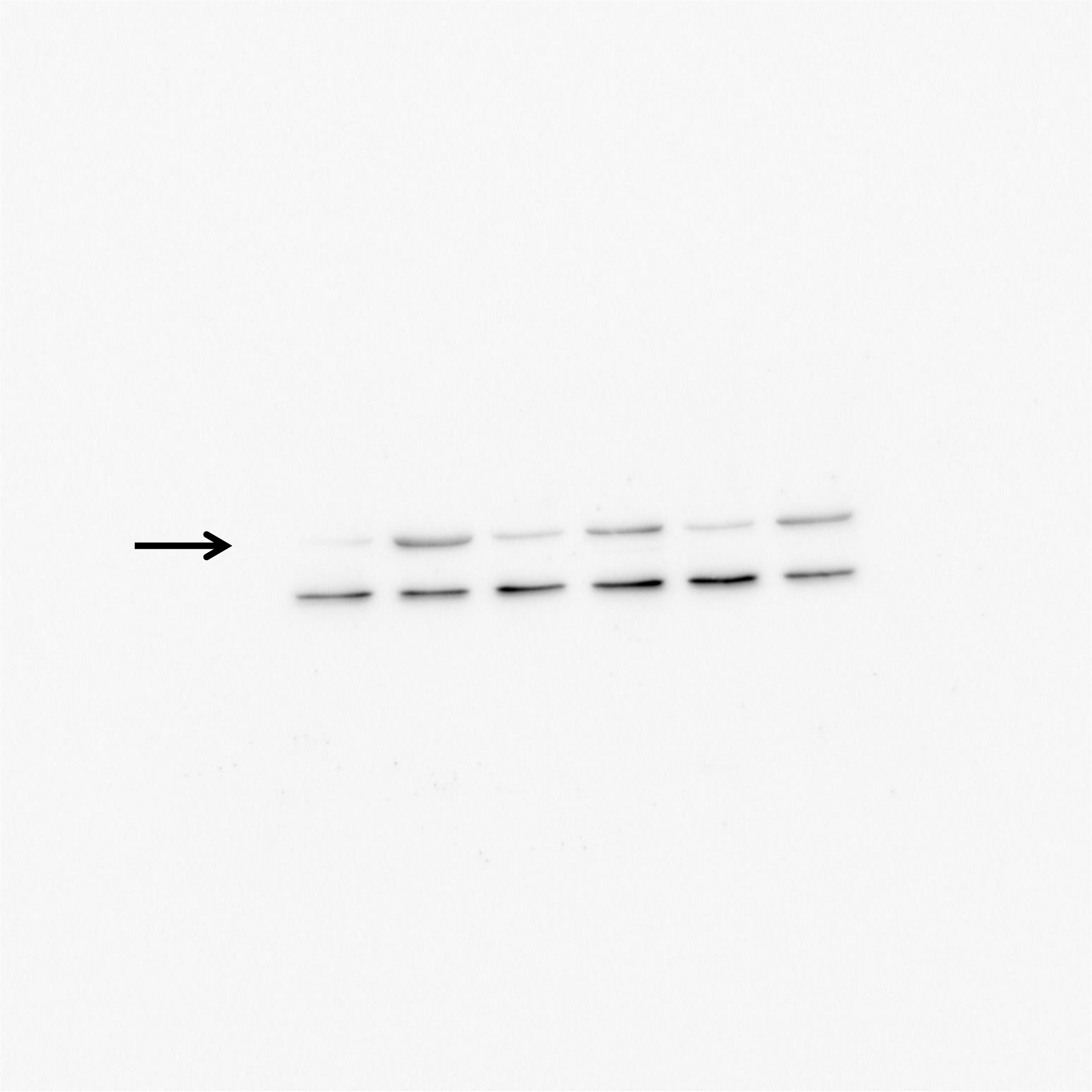

Figure 2 E1
Vinculin

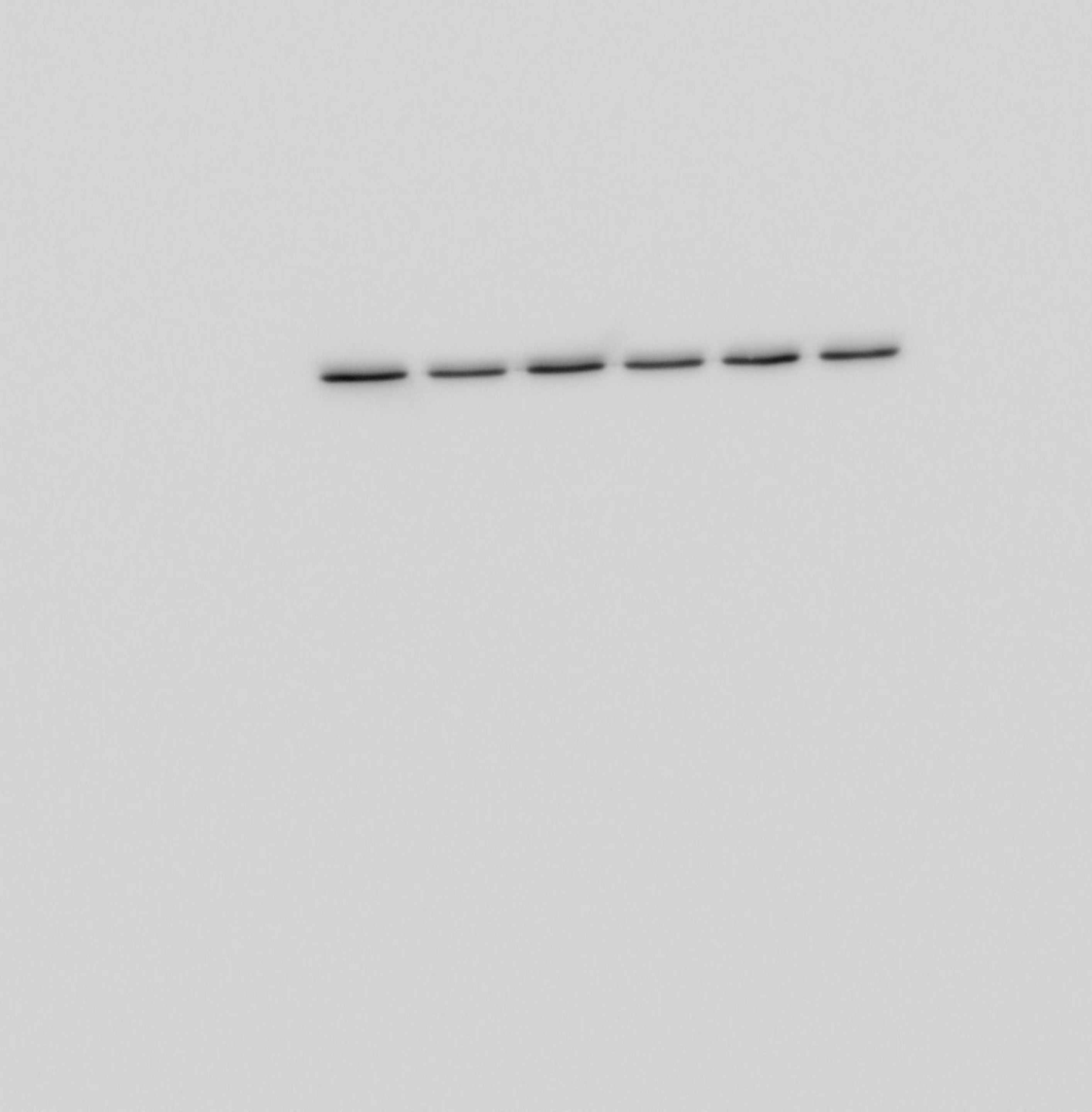

Figure 3 B1
NF-kB p65

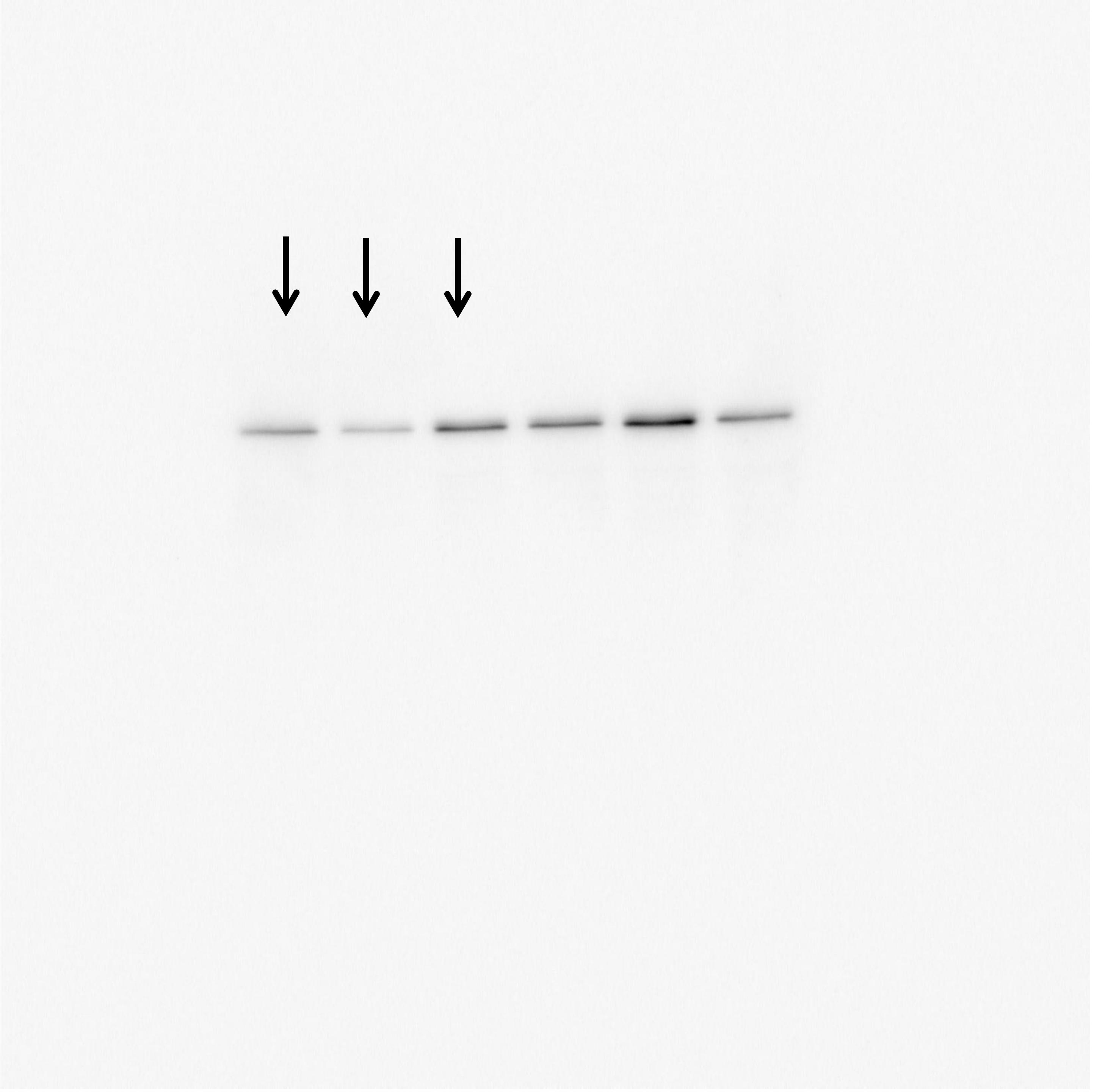

Figure 3 B1
Vinculin

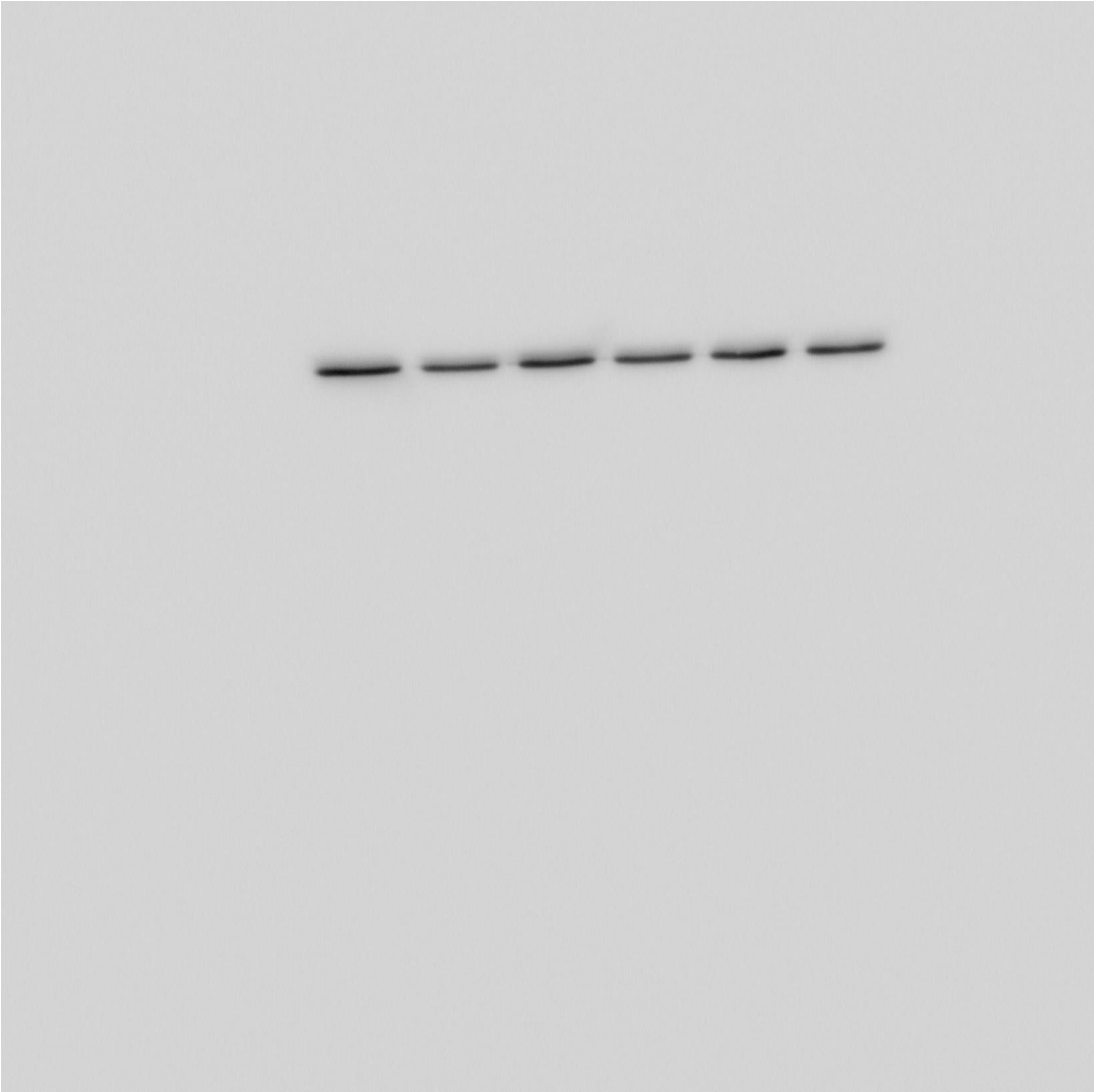

Figure 4 B1
Glo-I 6h and 12 h

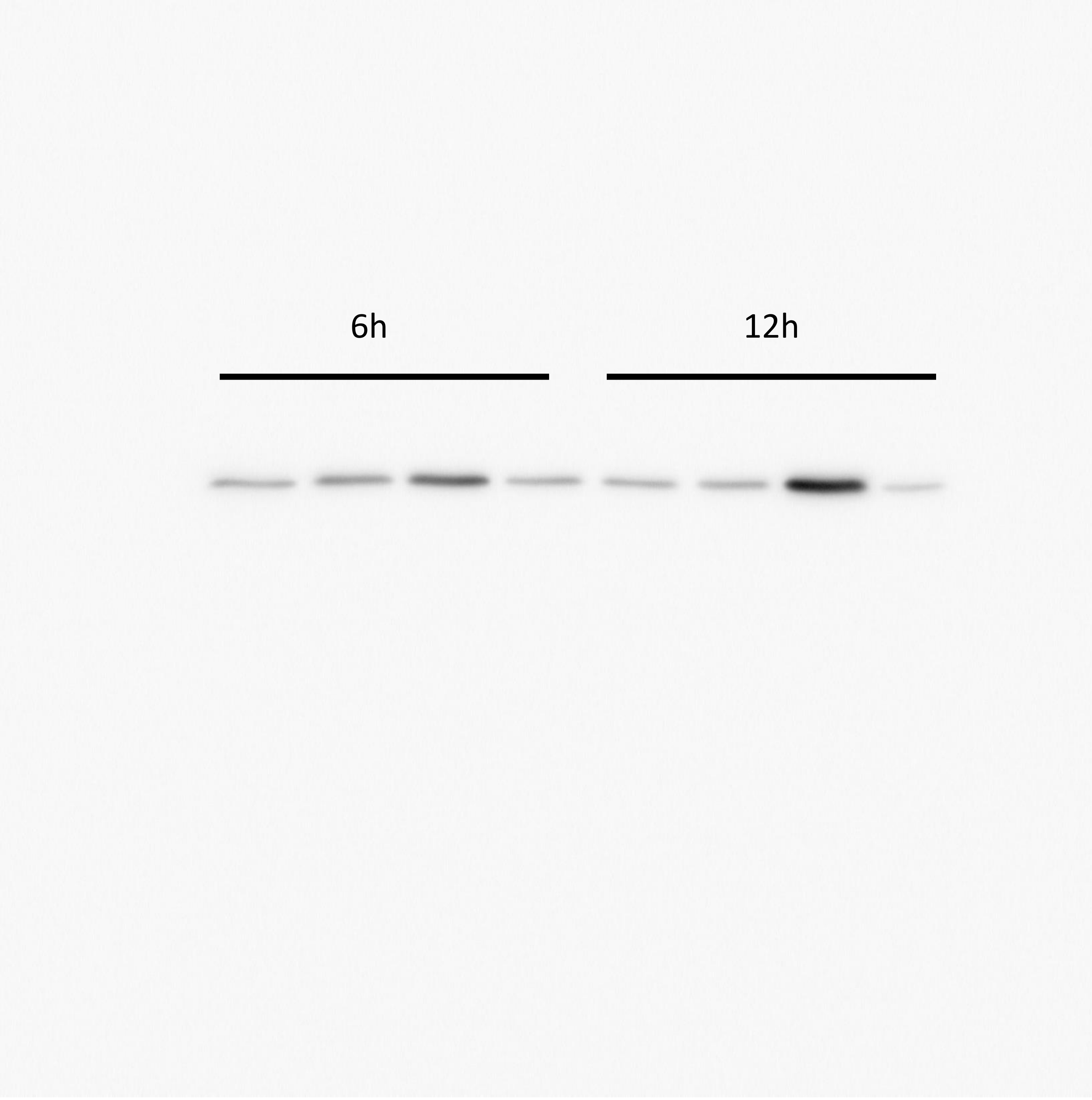

Figure 4 B1
Glo-I 24h

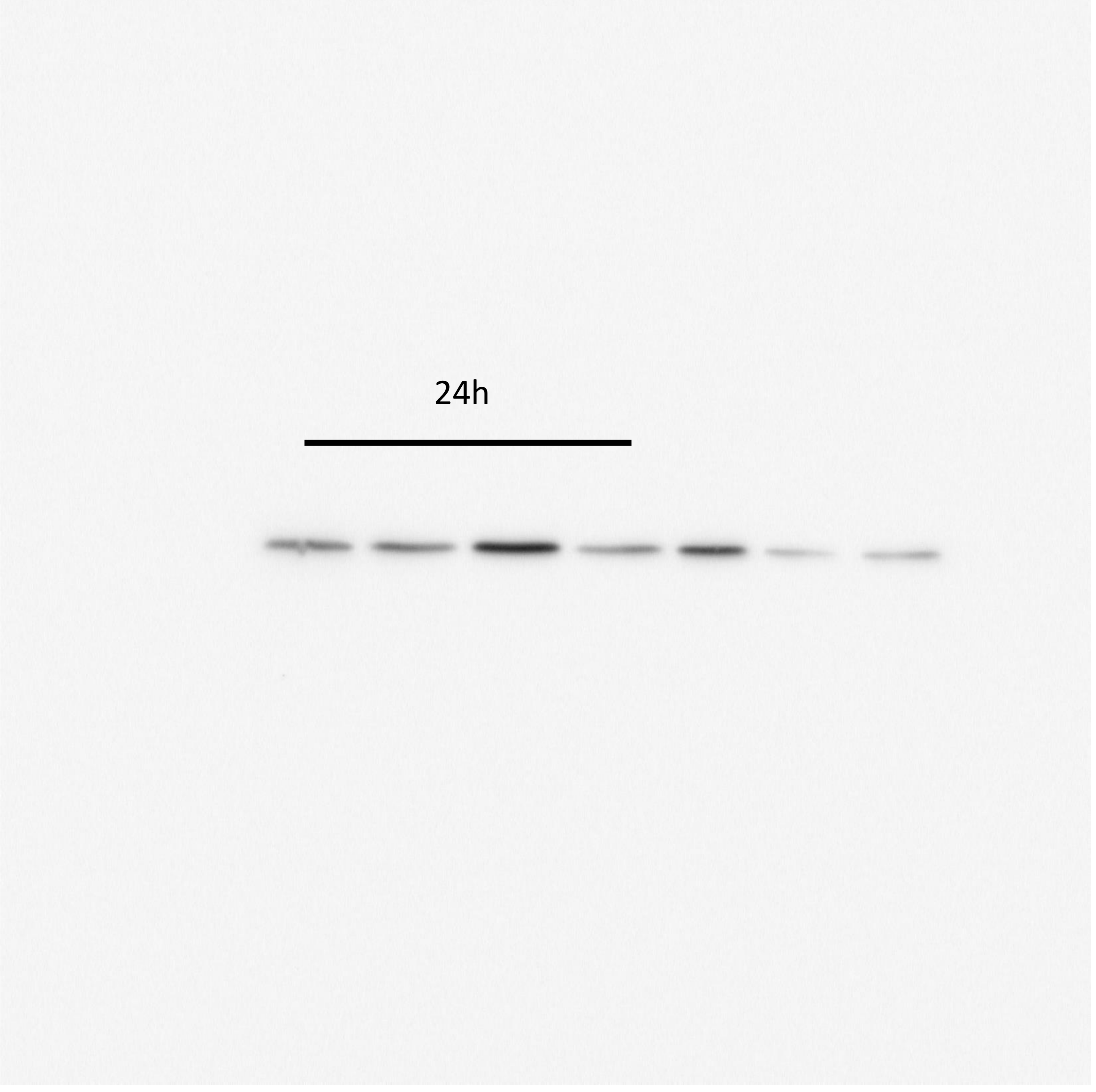

Figure 4 B1
Vinculin 6h and 12 h

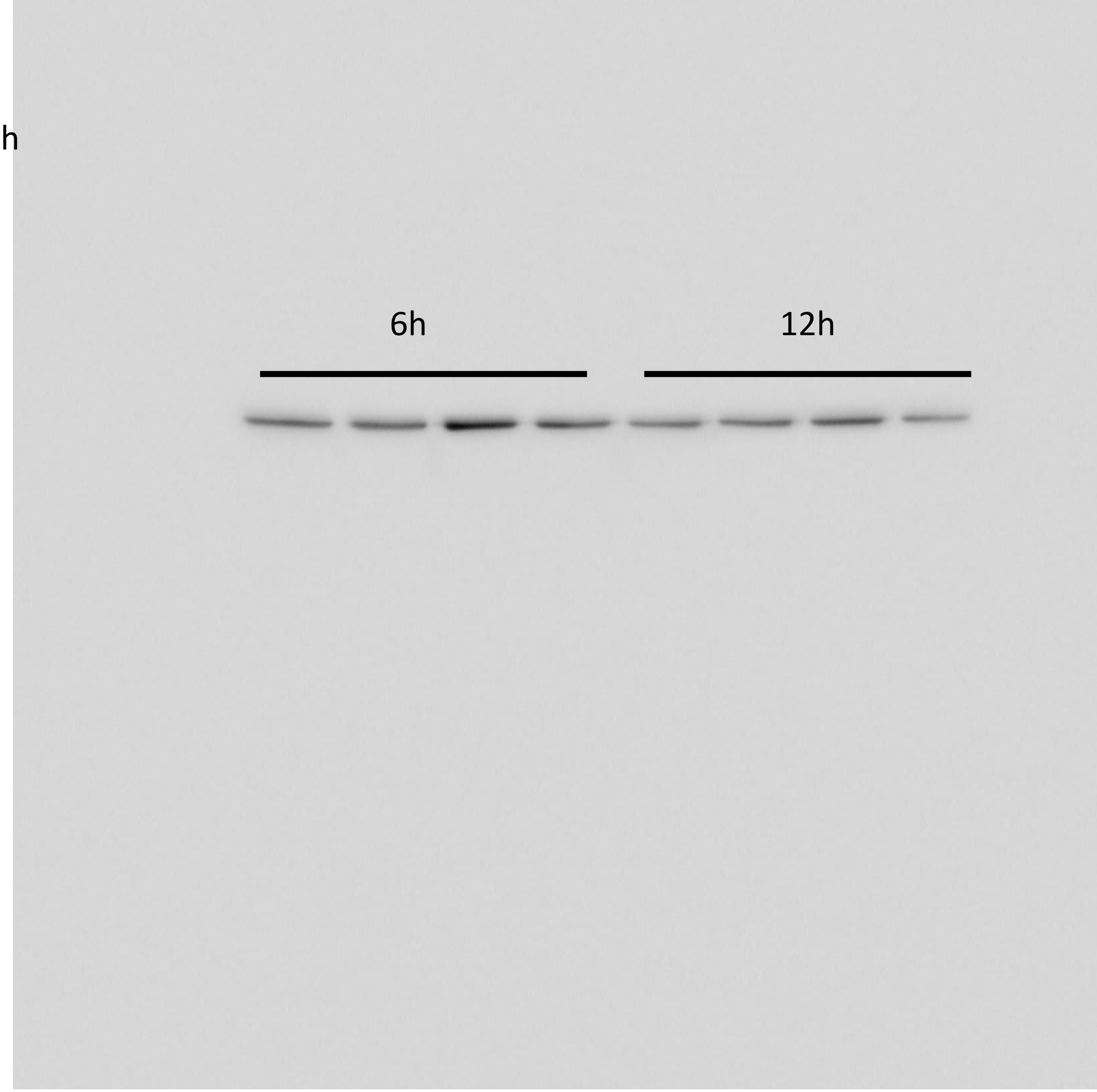

Figure 4 B1
Vinculin 24 h

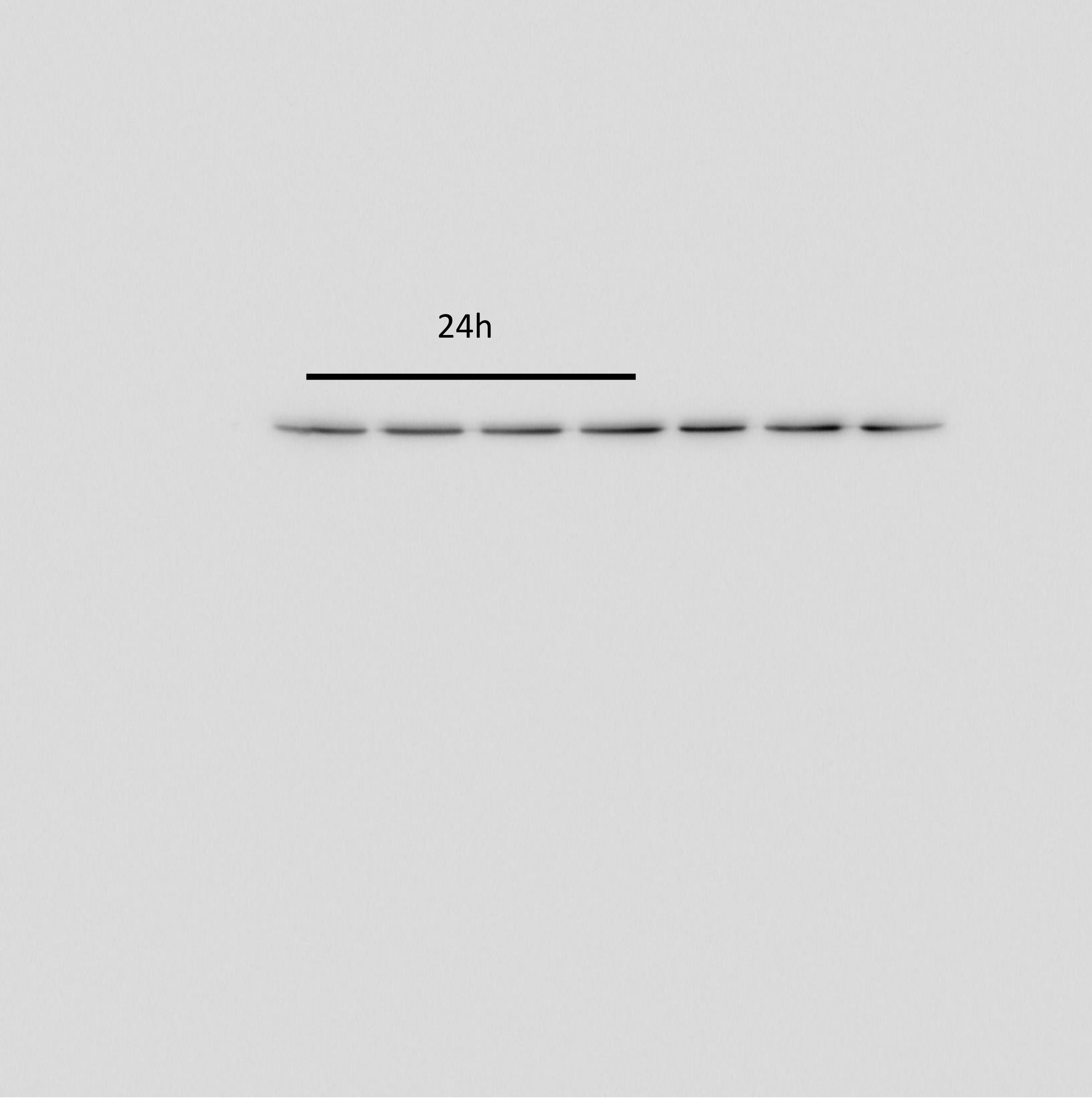

Figure 4 D1
Glo-I

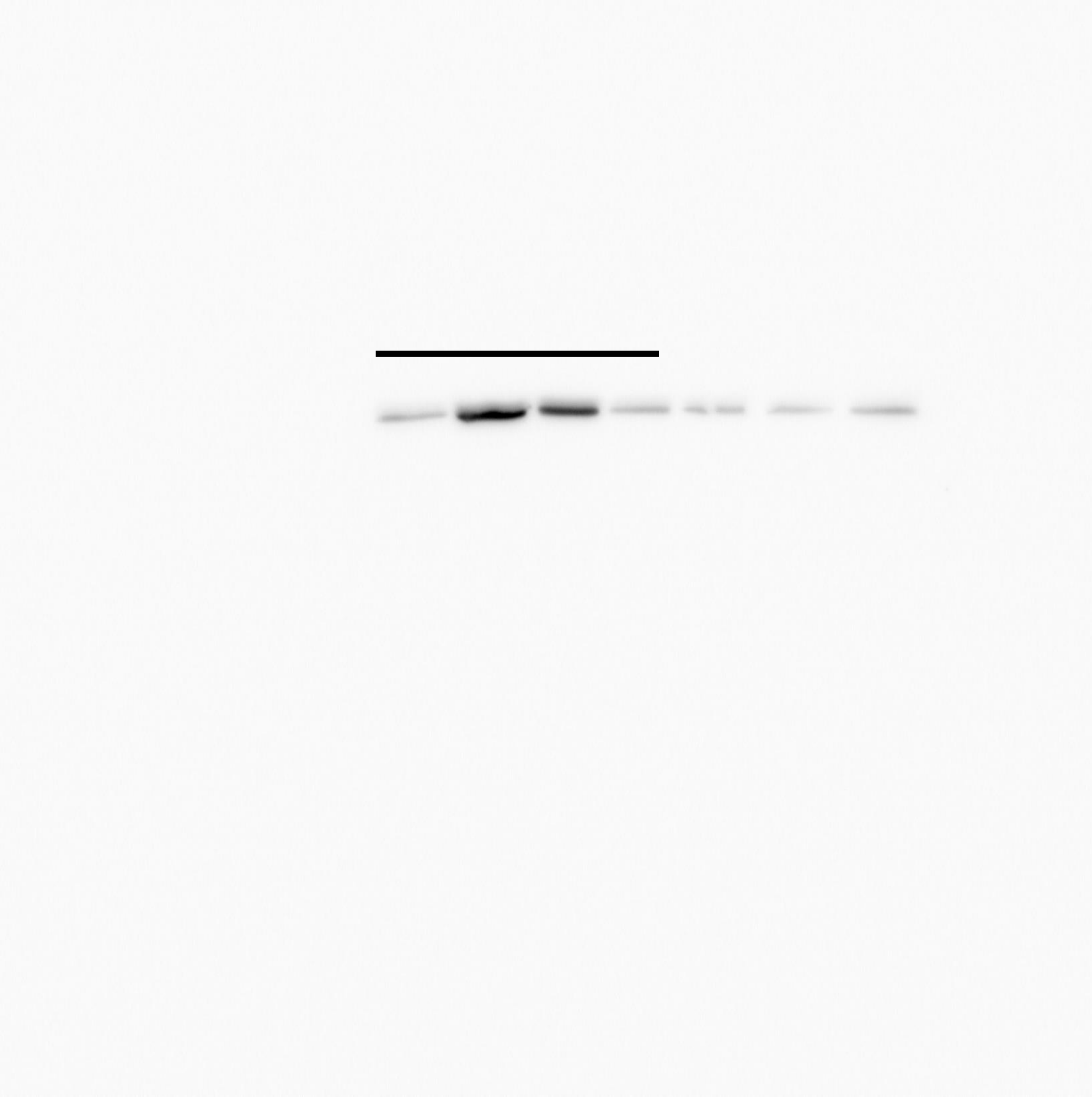

Figure 4 D1
Vinculin

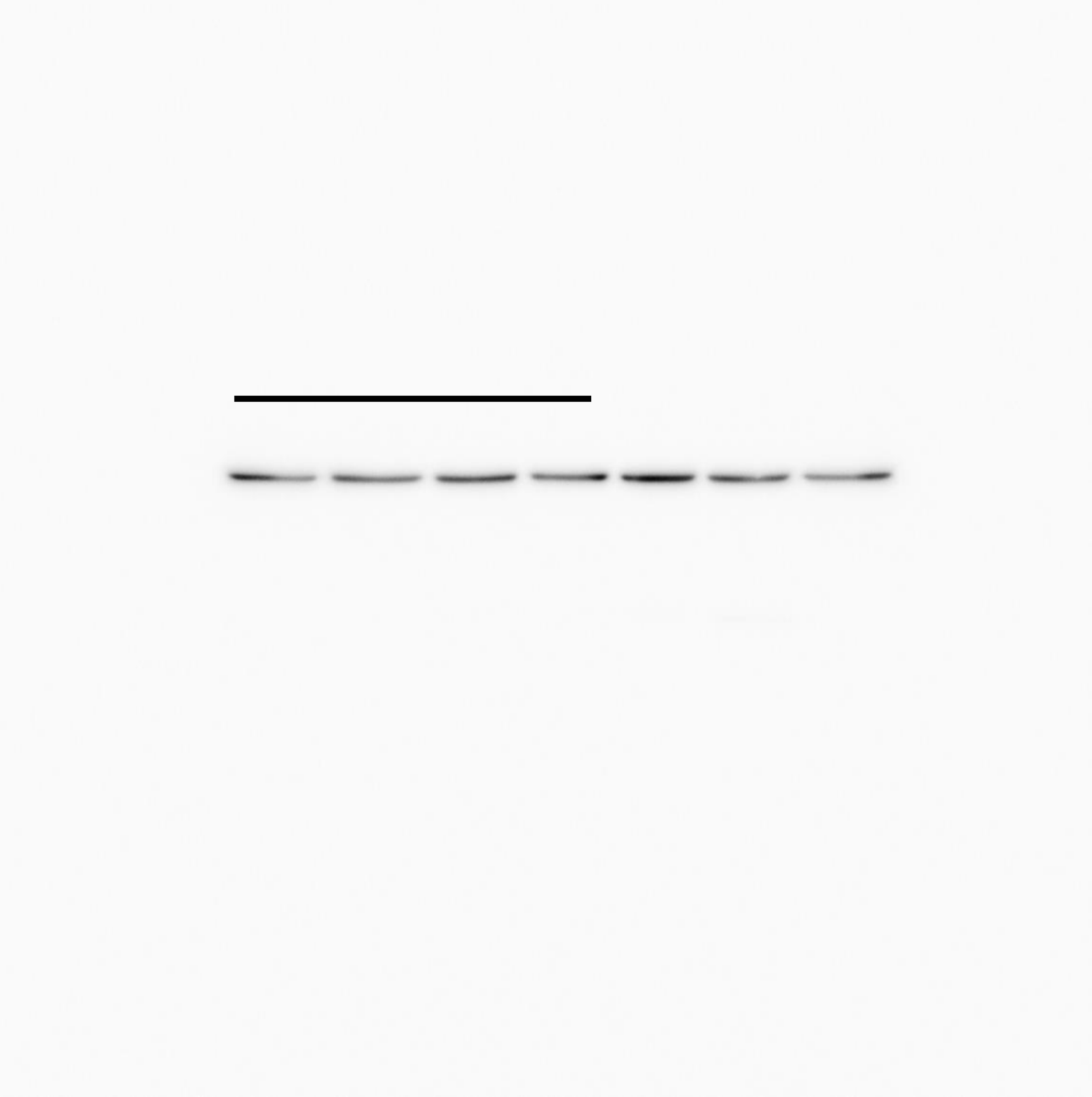

Figure 5 A1
Glo-I 24h

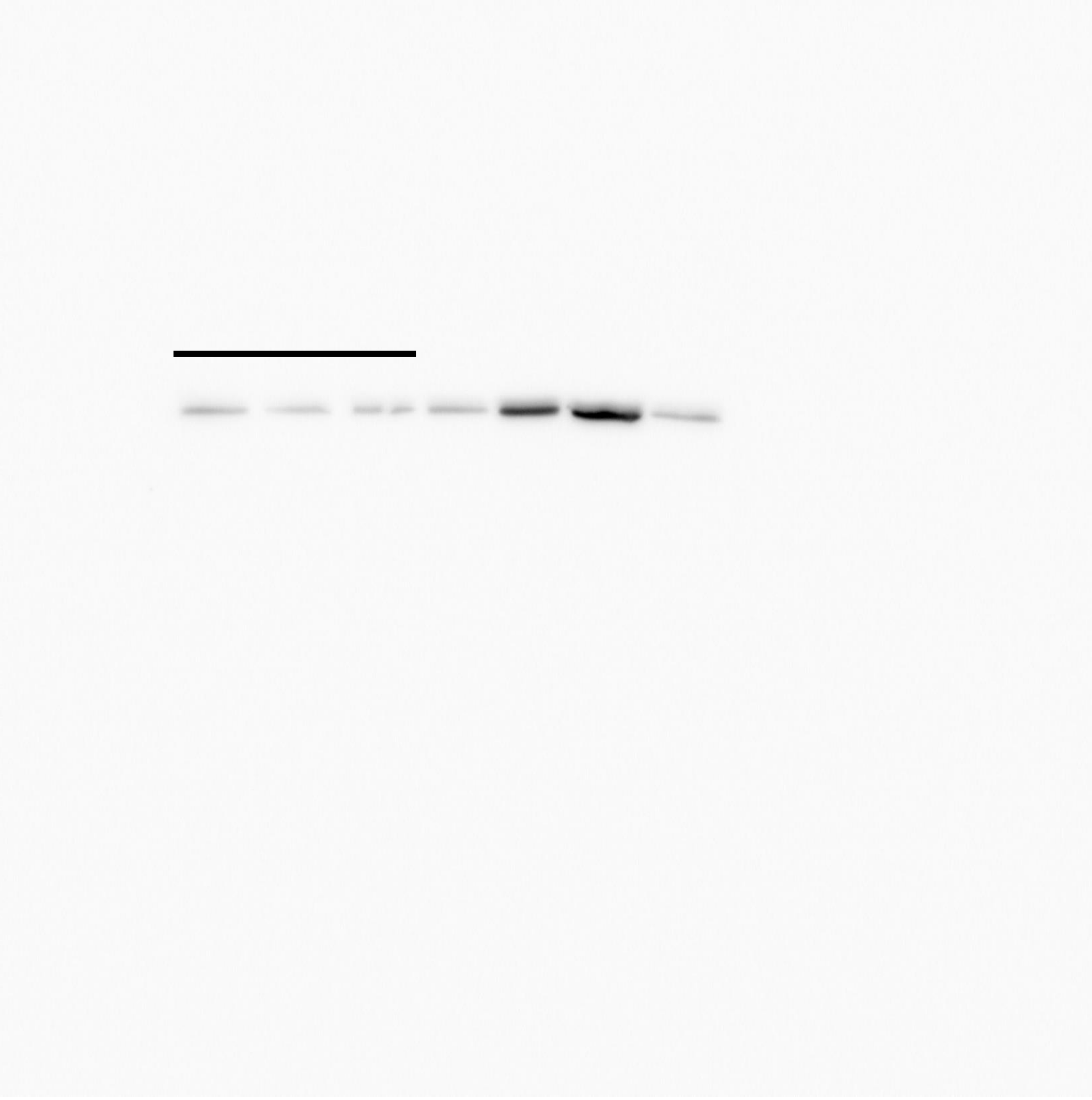

Figure 5 A1
Vinculin 24h

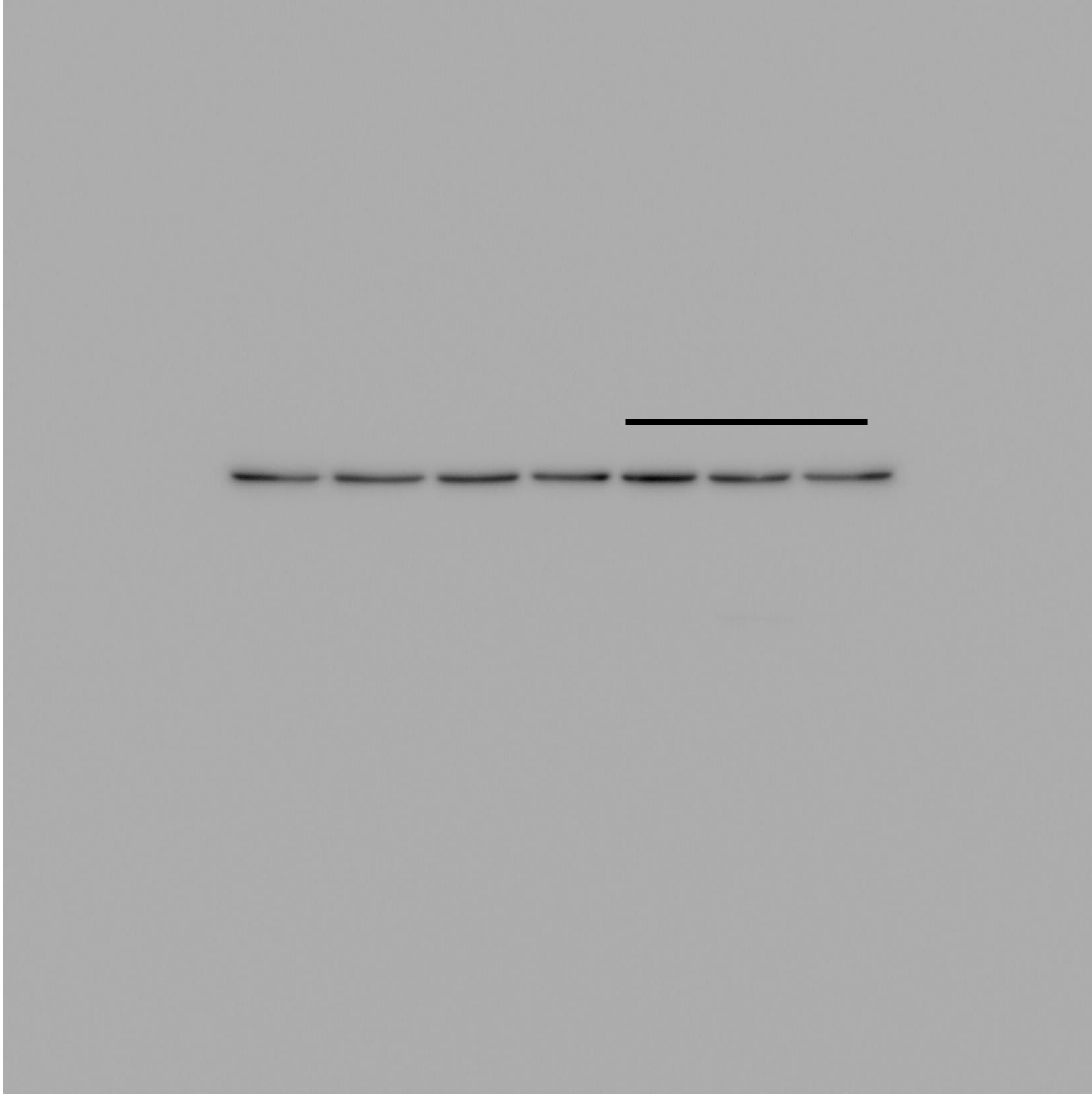

Figure 5 A1
Glo-I 48h

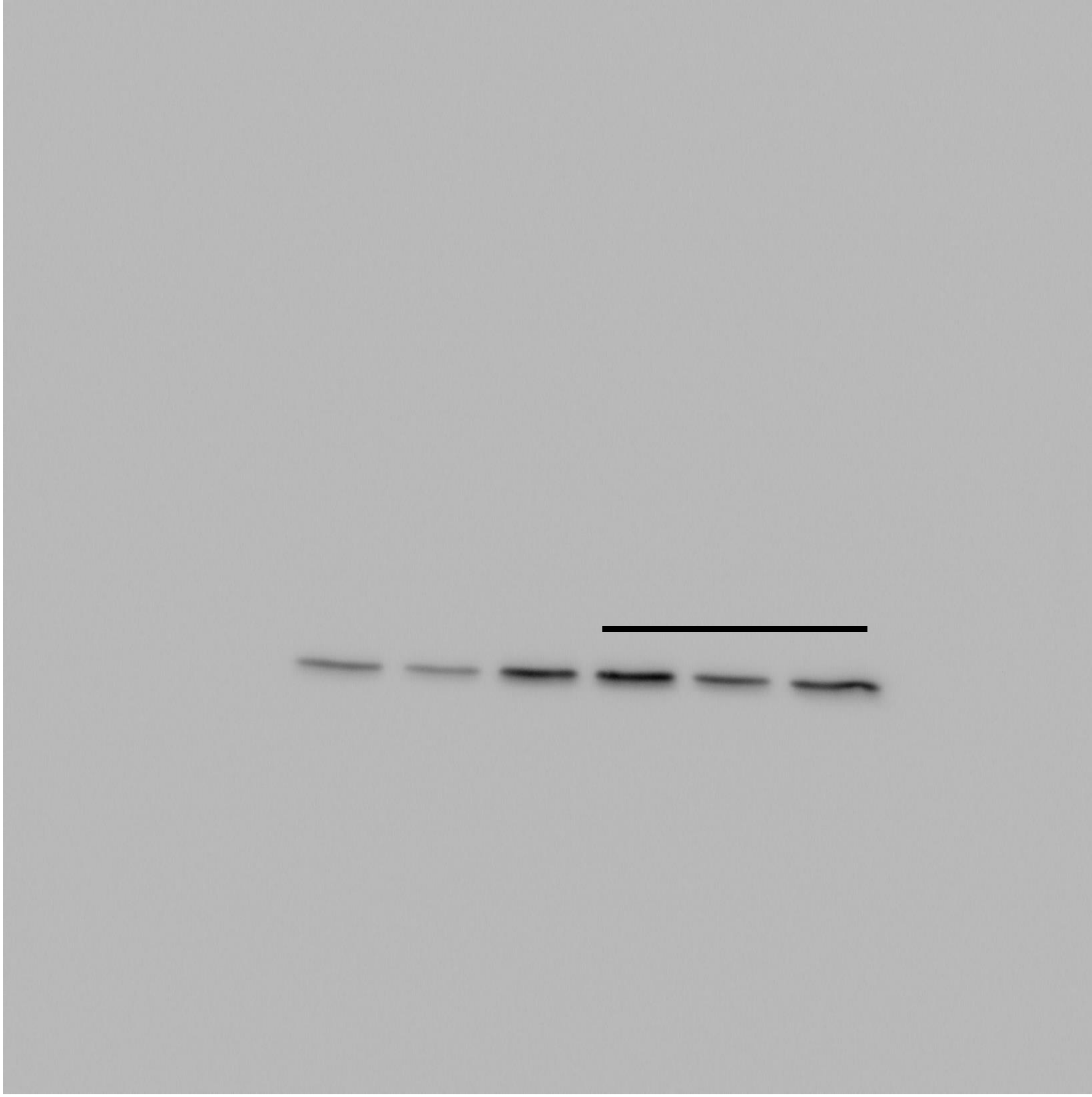

Figure 5 A1
Vinculin 48h

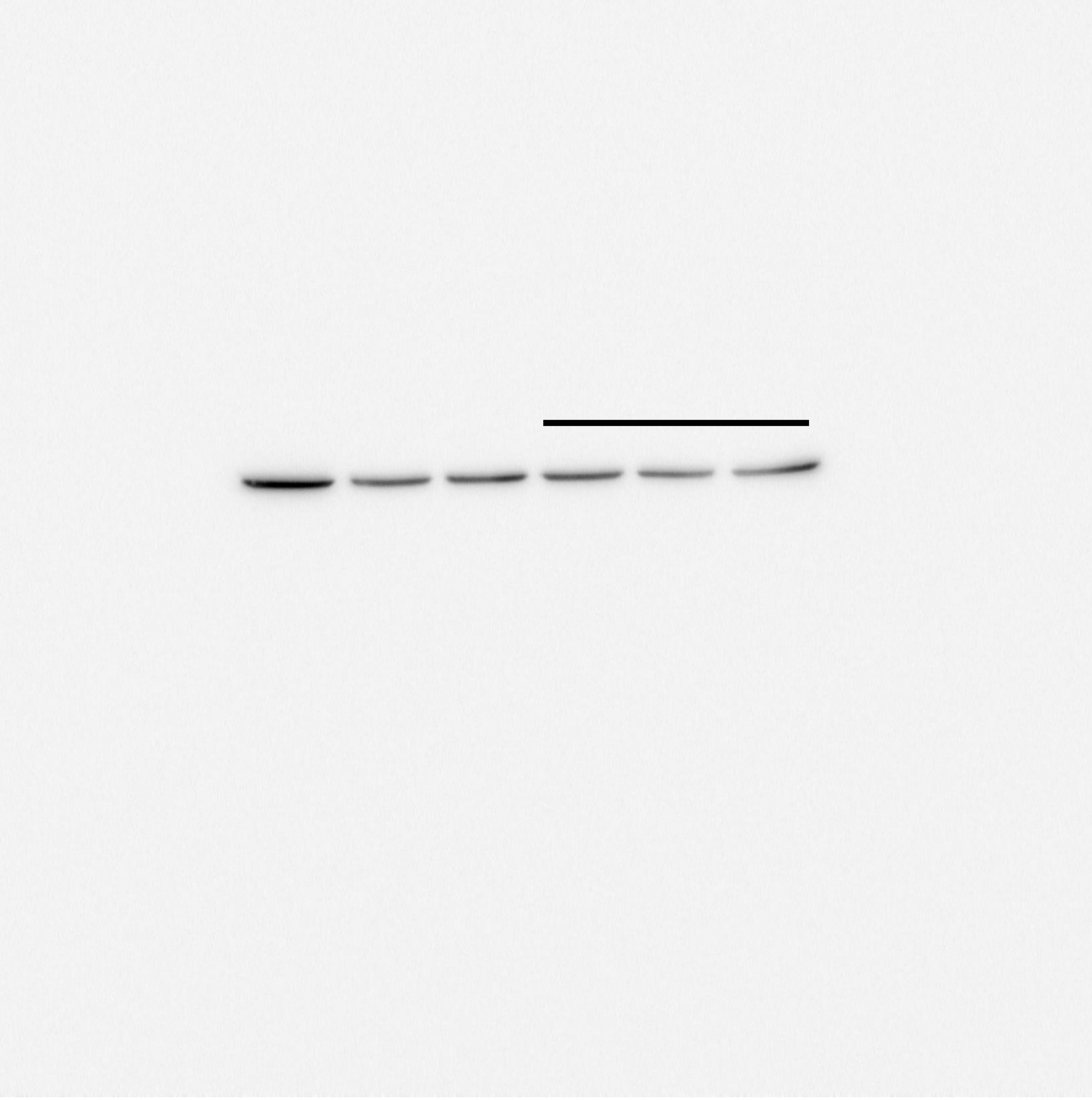

Figure 5 A1
Glo-I 72h

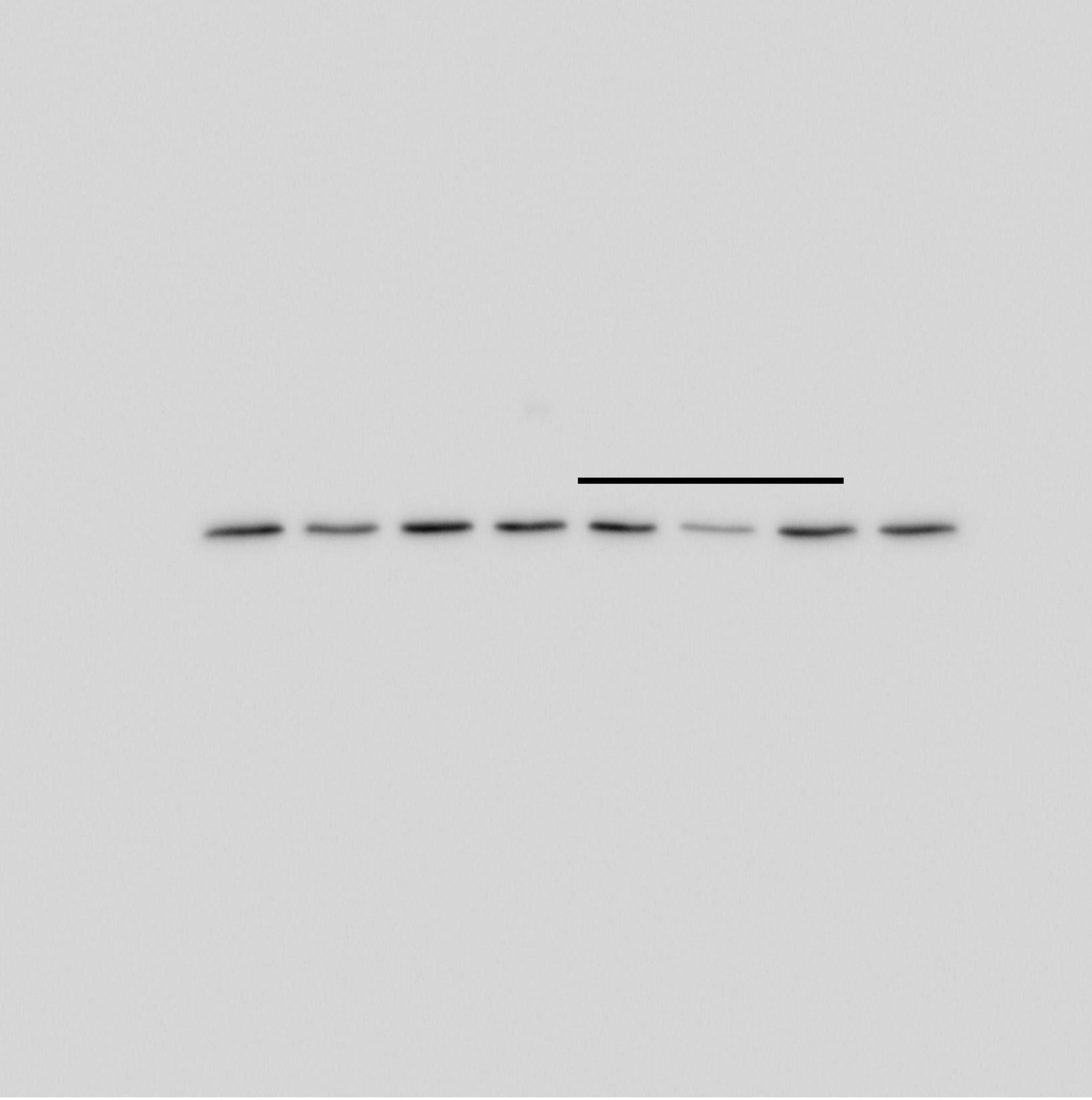

Figure 5 A1
Vinculin 72h

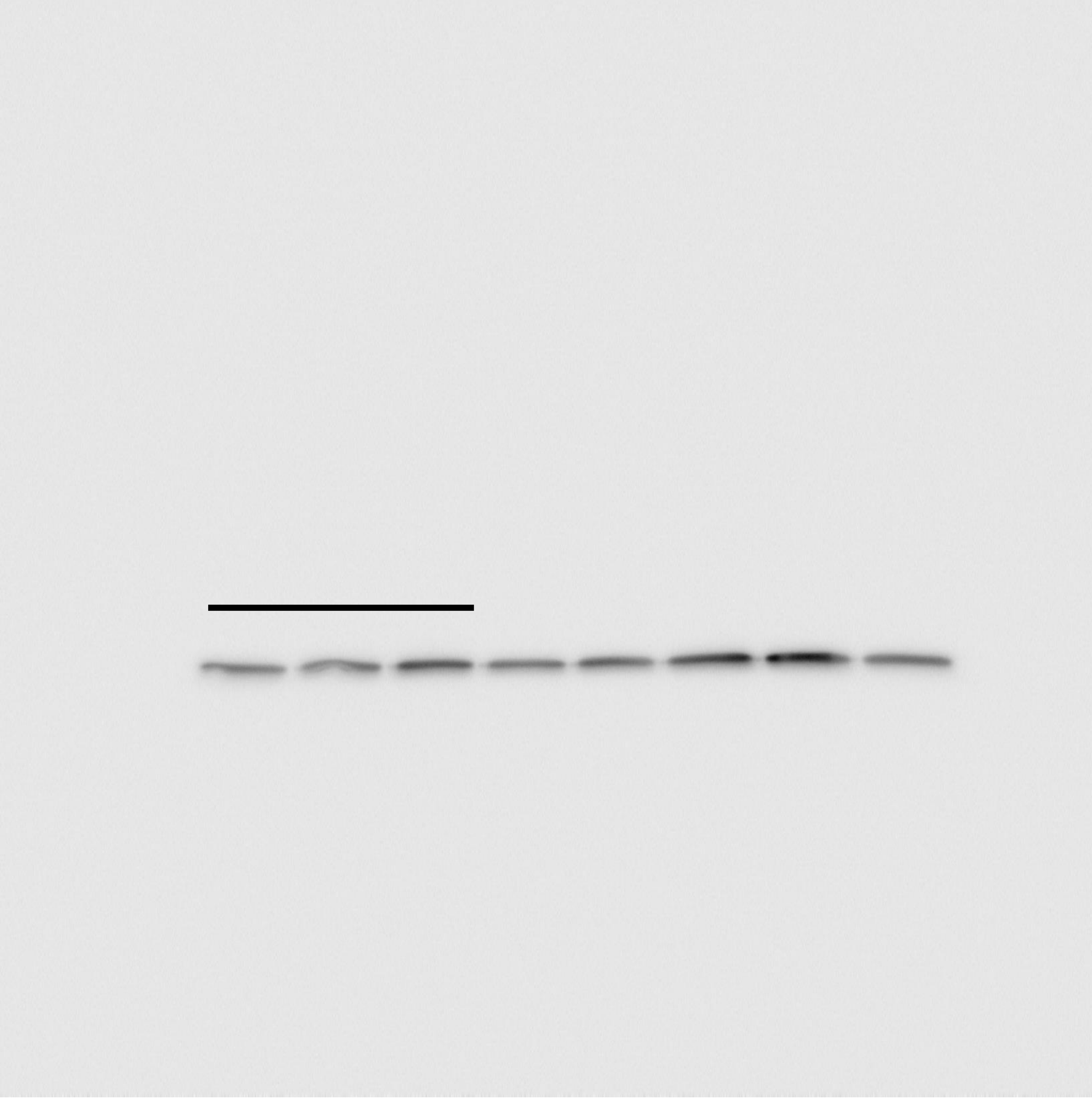

Supplement: S1 Fig — (PDF) [file pone.0242706.s001.pdf]
