# Supplementary material for: Pitfalls in AR42J-model of cerulein-induced acute pancreatitis
Source: PLoS One. 2021 Jan 25;16(1):e0242706. doi: 10.1371/journal.pone.0242706 (PMC7833168; doi:10.1371/journal.pone.0242706)
Supplement: Attachment — Submitted filename: 20_07_21 Rebuttal letter PlosOne MH.docx [file pone.0242706.s002.docx]

Zoltán Rakonczay Jr., M.D., Ph.D., D.Sc.

Academic Editor

PLOS ONE

PONE-D-20-13635: **“Pitfalls in AR42J-model of Cerulein-Induced Acute PancreatitisPitfalls in AR42J-model of Cerulein-Induced Acute Pancreatitis”**

Dear Dr. Rakonczay,

We thank you and the reviewer for your valuable comments and positive critiques. These comments have been very useful to us in making the necessary changes needed to improve the manuscript. We hope we have satisfactorily addressed them in the following paragraphs and that you will find the revised version of the manuscript suitable for publication. You will also find a PDF file containing the original images of blots and gels in the supplements.

Sincerely,

Marcus Hollenbach

***Editor***

*1. Please ensure that your manuscript meets PLOS ONE's style requirements, including those for file naming.*

We carefully checked PLOS ONE’s reference style guidelines and adapted the reference list.

*2. At this time, we ask that you please provide full and detailed methodology of the ELISA experiments carried out in your study in the Methods section, to ensure that other researchers can replicate and reproduce your experiments. In addition, please revise the names of the antibodies in your Western Blot section to include the prefix "anti-" (e.g. anti-alpha-amylase).*

We thank for this advice and provided full information regarding the ELISA experiments. The description of antibodies was revised as suggested.

*3. Please report your cell concentrations in scientific notation.*

We revised the manuscript as indicated.

*4. We note that you have included the phrase “data not shown” in your manuscript. Unfortunately, this does not meet our data sharing requirements. PLOS does not permit references to inaccessible data. We require that authors provide all relevant data within the paper, Supporting Information files, or in an acceptable, public repository. Please add a citation to support this phrase or upload the data that corresponds with these findings to a stable repository (such as Figshare or Dryad) and provide and URLs, DOIs, or accession numbers that may be used to access these data. Or, if the data are not a core part of the research being presented in your study, we ask that you remove the phrase that refers to these data.*

We thank for this advice and removed this statement as it is not essential for results or conclusion.

*5. PLOS ONE now requires that authors provide the original uncropped and unadjusted images underlying all blot or gel results reported in a submission’s figures or Supporting Information files. This policy and the journal’s other requirements for blot/gel reporting and figure preparation are described in detail at https://journals.plos.org/plosone/s/figures#loc-blot-and-gel-reporting-requirements and https://journals.plos.org/plosone/s/figures#loc-preparing-figures-from-image-files. When you submit your revised manuscript, please ensure that your figures adhere fully to these guidelines and provide the original underlying images for all blot or gel data reported in your submission. See the following link for instructions on providing the original image data: https://journals.plos.org/plosone/s/figures#loc-original-images-for-blots-and-gels. In your cover letter, please note whether your blot/gel image data are in Supporting Information or posted at a public data repository, provide the repository URL if relevant, and provide specific details as to which raw blot/gel images, if any, are not available. Email us at plosone@plos.org if you have any questions.*

We created a single PDF file named “S1_raw_images” and provided the original images of gels and blots as requested. The file was uploaded as supplementary material.

*6. Please amend either the title on the online submission form (via Edit Submission) or the title in the manuscript so that they are identical.*

The title was revised accordingly.

*7. Please include your tables as part of your main manuscript and remove the individual files. Please note that supplementary tables should be uploaded as separate "supporting information" files.*

Table 1 and 2 were included to the main manuscript.

***Reviewer #1:***

*In this manuscript, the authors presented AR42J cells as a cell-culture model of cerulein (CER)-induced acute pancreatitis as well as the conditions for the transfection and induction of proinflammatory markers in CER-treated AR42J cells. Unfortunately, there is no novelty in the study, because it is well known that these amphicrine cells should be treated by dexamethasone that favors their differentiation toward the exocrine phenotype and leads to increased secretion of amylase in response to the secretagogue cholecystokinin. AR42J cells have been also widely used as an “in vitro” model for the transfection to study the exocrine pancreas and it was described in published protocols and reviews (Logsdon CD, Moessner J, Williams JA, Goldfine ID. Glucocorticoids increase amylase mRNA levels, secretory organelles, and secretion in pancreatic acinar AR42J cells. J Cell Biol. 100:1200-1208, 1985; Gonzalez A S-CP, Salido GM (2011) Culture of pancreatic AR42J cell for use as a model for acinar cell function. In: The Pancreapedia: Exocrine Pancreas Knowledge Base 2011; Christophe J. Pancreatic tumoral cell line AR42J: an amphicrine model. Am J Physiol 266: G963-971, 1994; Mareninova, O.A. Orabi, Abrahim I. Husain, Sohail Z. (2015). Experimental acute pancreatitis: In vitro models. In: The Pancreapedia: Exocrine Pancreas Knowledge Base 2015).*

We thank the reviewer for this advice but clearly disagree with the reviewer’s opinion. Indeed, AR42J cells were used for decades as an *in-vitro* model for cerulein (CN)-induced pancreatitis. However, as demonstrated in detail in Table 1, the used conditions for that model dramatically vary between the published articles. Moreover, important details of the experimental setting often are missing. Our data therefore clearly describe the necessity of dexamethasone-treatment prior to CN-stimulation. In addition, different concentrations of CN and dexamethasone as well as incubation times were analyzed. Furthermore, we present an optimized protocol for successful overexpression and gene knockdown to maximize transfection efficacy.

*Reviewer #2: The article written by Hollenbach et al. provides a useful summary about the methodological difficulties that any researcher may face when working with AR42J cell line. The authors also created a guidance of the optimal experimental setup of this cell line that helps to follow good laboratory practice.*

We thank the reviewer for the positive critiques and we hope our article will help researches to optimize their own AR42J experimental setting.

*I have only a few reflections that should be clarified in the manuscript:*

*1. Abstract/ Results: “CN treatment resulted in increased TNS-alpha production but not secretion and did not influence IL-6.” The second part of this sentence should be corrected. Which characteristics of IL-6 is mentioned? Expression, IL-6 levels, production etc.?*

We thank the reviewer for this advice and corrected the corresponding sentence.

*2. Materials and methods / qRT-PCR: The details of cDNA construction should be mentioned here.*

We used the Qiagen QuantiTect one stop qRT-PCR kit. This kit allows the easy and fast qRT-PCR without necessity of checking cDNA. RNA was used as described in the manufacturers’ manual. We updated the information in this section regarding the used PCR protocol.

*3. Materials and methods / Glo-I plasmid generation: Primer orientation should be indicated (5’ – 3’), and heat shock temperature of E.coli transformation should be mentioned.*

This section of material and methods was revised and the requested information provided.

*4. Figure 2C. It is mentioned that LPS was not able to induce a secretion of amylase by AR42J. However, it seems that LPS treatment inhibited the increase of amylase levels after dexamethason pretreatment and CN treatment. According to the Figure2C amylase levels decreased if cells were treated by LPS and CN together compared to CN only treatment. Could you please explain this observation? Is it possible that this figure is inaccurate?*

We are very thankful for this advice. Indeed, fig. 2C was incomplete. Our data showed that LPS treatment alone was not able to induce amylase secretion. In addition, co-treatment of LPS and CN did not further influence the release of amylase compared to CN alone. We corrected fig. 2C and clarified the statement in the manuscript.

*Reviewer #3: Hollenbach et al. present a methodological paper on the usability of AR42J cells for investigation of some aspects of the pathophysiology in acute pancreatitis. They show that caerulein can be used in combination with dexamethasone to induce amylase and cytokine secretion. They also investigated the expression of nf-κΒ and transfected cells with a plasmid containing glyoxalase-I or glyoxalase-I siRNA. The authors conclude that AR42J cells are a useful in-vitro model for caerulein induced acute pancreatitis when considering some experimental conditions. The paper covers an important topic when investigating experimental pancreatitis. The methods are clearly presented and the results contain novelty.*

We thank the reviewer for the positive evaluation of our manuscript. Indeed, we hope to provide evidence and guidance for researches that are faced with the AR42J-model of CN-induced pancreatitis.

*There are some aspects the authors need to consider:*

*1.) In the images of figure 1 and figure 4, A1 and A3 the brightness of AR42J cells should be adjusted.*

We thank for this suggestion of optimized brightness of the corresponding images.

*2.) The authors should mention in the introduction why the investigate Glyoxalase-I expression in AR42J cells.*

The Glyoxalase-System demonstrates a main research interest for many years in the authors’ lab. Ongoing studies analyze the role of Glyoxalas-I in acute and chronic pancreatitis as well as pancreatic cancer *in vivo* and *in vitro*. This was highlighted in the introduction section.

*3.) It should be mentioned in the results section (p. 11-12), which concentration of CN leads to the highest amylase secretion.*

It was now stated at results section that 100nM CN was used for the following experiments as this concentration induced highest amylase release.

*4.) The figures should be arranged in a numerical order starting with figure 1. Here the authors start with figure 6A (p. 11).*

We agree with the reviewer’s suggestion and referred in the corresponding paragraph to sections below. Now the figures order follows the results presentation in the manuscript.
